# Male kidney allograft recipients at risk for urinary tract infection?

**DOI:** 10.1371/journal.pone.0188262

**Published:** 2017-11-16

**Authors:** Gerold Thölking, Katharina Schuette-Nuetgen, Thomas Vogl, Ulrich Dobrindt, Barbara C. Kahl, Marcus Brand, Hermann Pavenstädt, Barbara Suwelack, Raphael Koch, Stefan Reuter

**Affiliations:** 1 Department of Medicine D, Division of General Internal Medicine, Nephrology and Rheumatology, University Hospital Münster, Münster, Germany; 2 Institute of Immunology, University of Münster, Münster, Germany; 3 Institute of Hygiene, University of Münster, Münster, Germany; 4 Institute of Medical Microbiology, University Hospital Münster, Münster, Germany; 5 Institute of Biostatistics and Clinical Research, University of Münster, Münster, Germany; Medizinische Universitat Graz, AUSTRIA

## Abstract

**Background:**

Urinary tract infection (UTI) is the most common infection after renal transplantation (RTx). Although female sex is a well-known risk factor for the development of UTI after RTx, the role of the donor sex in this context remains unclear.

**Methods:**

In this case control study 6,763 RTx cases were screened for UTI when presenting at our transplant outpatient clinics. 102 different RTx patients fulfilled the inclusion criteria and were compared to 102 controls. Data on renal function was prospectively followed for 12 months. Results were compared to a previous RTx cohort from our transplant center. Additionally, we assessed the immunological response of leukocytes from 58 kidney recipients and 16 controls to lipopolysaccharide stimulation.

**Result:**

After identification by univariate analysis, multivariate logistic regression analysis indicated female sex, minor height, advanced age and male kidney allograft sex to be associated with the occurrence of UTI after RTx. Female recipients who received male grafts had the best renal function 12 months after presentation. However, leukocyte response of recipients to lipopolysaccharide was impaired irrespective of donor and recipient sex to the same extend.

**Conclusions:**

We conclude from our data that male kidney allografts are associated with the occurrence of UTI after RTx but did not influence the response of leukocytes to lipopolysaccharide. Further prospective studies are needed to identify the underlying mechanisms of higher male kidney donor dependent UTI.

## Introduction

Urinary tract infection (UTI) is the most frequent infection occurring in more than one third of patients during the first year after renal transplantation (RTx) [1].

Host factors like sexual behavior include modifiable aspects potentially influenced by patients to reduce the risk for UTI. Non-modifiable risk factors provide a possible target for therapeutics or risk profiling [2]. In a recent meta-analysis Wu et al. observed that female sex, older age of the recipient, long duration of catheter, acute rejection episodes, and cadaveric donor are associated with a higher risk of UTI [1]. Given that UTI causes morbidity and is considered as a potential risk factor for inferior outcome after RTx, strategies to reduce UTI are needed.

Currently, the modification of potential risk factors is limited to a few parameters such as immunosuppression or insertion of bladder catheter postoperatively. As precautions like prophylactic antibiotic therapy [3] and removal of ureteral and Foley catheter early after RTx can be performed, the identification of new UTI risk factors might assist in a better risk stratification of patients.

In a recently published study, we found a strong association between the metabolism rate of the calcineurin inhibitor (CNI) tacrolimus (Tac) and the occurrence of BK virus (BKV) infections after RTx [4]. A fast Tac metabolism rate had a negative effect on the BKV infection rate. The Tac metabolism rate was defined as the Tac blood trough concentration (C) divided by the daily dose (D). Therefore, we tested the hypothesis if Tac metabolism is also associated with the presence of UTI.

Further, the donor sex might be a risk factor for UTI. Efforts have been made to characterize the impact of the donor sex on the outcome after RTx. It was shown that female recipients of male kidney transplants had an increased risk for adverse outcomes. This was attributed to the H-Y antigen mismatch/H-Y effect [5, 6]. Therefore, donor sex might have an immunological impact on the recipient. Additionally, Antus et al. concluded from an animal study that sex hormones impact on graft survival [7] and testosterone was shown to promote renal damage in mice and men [8, 9]. However, (urinary) infection rates have not been investigated in these studies. In contrast, a role for sex hormones on female UTI rate is well known [10].

In addition, differences in male and female urine proteomic patterns have been recently identified which might also have an influence on urinary bacterial proliferation and inflammatory processes [11, 12].

The evidence of donor sex influence on the renal outcome in different ways after RTx, encourages us to correlate the donor sex with the occurrence of UTI.

## Patients and methods

### Study population

In this case-control study, we included 102 UTI patients who presented at our transplant outpatient clinic between August 2012 and September 2014 and had an UTI at this visit (symptomatic and asymptomatic). UTI was considered as clinically relevant in case of pyuria, ≥100,000 colony-forming units (CFU)/ml urine or symptoms of bladder infection and more than 1000 CFU/ml urine [13]. Symptomatic bladder infection was characterized by increased frequency, urgency, dysuria, or suprapubic pain. Urine culture was sent in case of pyuria or symptoms of bladder infection. Every patient was included only once even in case of relapse or reinfection. We included only patients with a tacrolimus (Tac)-based immunosuppressive regimen containing immediate release-Tac, in combination with mycophenolate mofetil, mycophenolic sodium or everolimus ± prednisolone (≤ 20 mg/day). All patients received an induction therapy with basiliximab. We excluded patients with active malignancy, chronic infection, and pregnant or breast-feeding women. Among the enrolled patients, RTx was performed between April 1987 and August 2014. Data of UTI patients was compared with a selected control group of 102 patients who had no UTI at their presentation visit (August 2012 until September 2014) and were chosen based on a comparable time span since RTx (RTx between March 1990 and June 2013).

Patients’ and donors’ characteristics were taken retrospectively from the electronic patient records. Drug doses, blood levels, estimated glomerular filtration rate (eGFR) values, death events and data indicative of infection such as C reactive protein (CRP), leukocytes and germs were collected prospectively. Former occurrences of UTI since RTx and following UTIs after recruitment visit were not recorded. Data of all patients was anonymized prior to analysis. Written informed consent was obtained from all participants in case of tissue sampling as well as recording their clinical data and use in anonymized analysis. All methods, experiments on humans and the use of human tissue samples in this study were performed in accordance with the current transplantation guidelines and the Declarations of Istanbul and Helsinki. All methods in this study were approved by the local ethics committee (Ethik Kommission der Ärztekammer Westfalen-Lippe und der Medizinischen Fakultät der Westfälischen Wilhelms-Universität, No. 2014-381-f-N and No. 2011-400-fs).

In a second step, study results were compared to previous data from a different RTx cohort from our transplant center (63 with UTI and 59 controls without UTI) [14]. In this cohort, the presentation at our transplant office was between May 2011 and October 2015. Patients were excluded from that cohort if they had been included in the present study cohort.

For the immunological stimulation tests, leukocytes of 16 apparently healthy controls (5 male, mean age 39.6±13.4 years, range 20–63 years) and 58 stable RTx patients (34 male, mean age 53.6±11.9 years, range 25–78 years) without active cancer or infection receiving an immunosuppressive therapy including immediate release-Tac ± mycophenolate mofetil, mycophenolate sodium or everolimus ± prednisolone were used.

### Cell culture and leukocyte stimulation

Whole blood was obtained in EDTA containing tubes under sterile conditions. 300 μL whole blood were added to 300 μL RPMI 1640 culture medium (PAA Laboratories, Pasching, Austria) supplemented with 2 mM L-glutamine, 50 μg/mL penicillin/streptomycin, 5 mM HEPES, 10 μM mercapto-ethanol and 0.1% fetal calf serum, and seeded in 24-well plates (Greiner, Nürtingen, Germany) at 37°C (5% CO_2_/95% air atmosphere). For stimulation, 1 ng/mL lipopolysaccharide (LPS, Sigma-Aldrich, Taufkirchen, Germany) was added to culture medium. Supernatants were collected after 24 h and stored at -20°C until analyses. LPS was chosen for stimulation because of its important role for immunological recognition of and response to uropathogens, i.e. *E*. *coli* [2]. Levels of pro-inflammatory interleukin-1ß (IL-1β), IL-8, in the culture supernatant were measured by enzyme-linked immunosorbent assay (ELISA) according to manufacturer‘s protocol and the alarmin S100A8/S100A9 was measured by a home-made ELISA as described in [15].

### Microbiological procedures

Native urine was sent to the Institute of Medical Microbiology of the University Hospital for analysis. 10 microliters of urine were streaked on Columbia blood agar (Becton Dickinson, Heidelberg, Germany) for quantitative bacterial analysis. A loop of urine was streaked on Columbia blood and McConkey (Becton Dickinson) agar for bacterial culture. Isolated bacteria were differentiated by MALDI-TOF analysis (Bruker Daltonics, Bremen, Germany). Susceptibility testing was performed via VITEK 2 system (bioMérieux, Nürtingen, Germany).

### Clinical chemistry

Whole blood was analyzed for CRP, white blood cell count and creatinine (enzymatic assay; Creatinine-Pap, on a Roche Diagnostic analyzer, Modular P, Roche diagnostics, Mannheim, Germany). Tac trough levels were assessed by LC-MS/MS. Kidney function was determined by eGFR calculation using the CKD-EPI formula at time of study inclusion and after a 12 months follow-up.

### Tacrolimus metabolism rate

The C/D ratio was calculated by dividing the drug blood trough concentration (C) by the corresponding daily Tac dose (D). The “non-weight-adjusted C/D ratio” and “weight-adjusted C/D ratio” were calculated as follows:

$$C/Dratio(ng/mL*1/mg)=\frac{bloodTactroughlevel(ng/mL)}{dailyTacdose(mg)}$$ (1)

C/Dratio(ng/mL*1/mg*KG−1)=bloodTactroughlevel(ng/mL)dailyTacdose(mg/KG) (2)

### Statistical analysis

Statistical analyses were performed using IBM SPSS^®^ Statistics 24 for Windows (IBM Corporation, Somers, NY, USA) and SAS software, version 9.4 of the SAS System for Windows (SAS Institute, Cary, NC, USA). Inferential statistics like p-values and confidence intervals were intended to be exploratory, not confirmatory. Therefore, neither global nor local significance levels were determined, and no adjustment for multiplicity was applied. P-values ≤0.05 were considered as statistically noticeable.

Standard univariate statistical analyses were applied. Categorical variables are shown as absolute and relative frequencies. Fisher’s exact tests were used to quantify the evidence between categorical variables. Normal-distributed continuous variables are shown as mean ± standard deviation. Not normal-distributed continuous variables are reported as median [minimum–maximum]. Groups were compared using Student’s t-tests for normally distributed data, Mann–Whitney U tests for non-normal data and Fisher's exact tests for categorical variables. Additionally, multivariable logistic regressions were applied to estimate the influence on the occurrence of UTI at date of inclusion and to adjust for confounders. Age at UTI (years), height (cm), log-transformed Tac C/D ratio at UTI (weight-adjusted), sex (male/female), donor sex (male/female), living donor transplantation (yes/no), and ESP (yes/no) were included as influencing factors. Results are presented as odds ratios (OR) and corresponding 95% confidence intervals (95% CI). Based on the logistic regression estimates, prediction probabilities for UTI at date of inclusion were calculated. ROC curves and area under the curve (AUC) were used to quantify the diagnostic accuracy of the model prediction. For comparison of predicted and observed UTI rates, patients were classified into quintiles according to their individual predicted probability to suffer from UTI. Boxplots were used for visualization.

For sensitivity analysis, the same logistic regression model was applied on the data from the previous cohort. Additionally, the parameter estimates based on the actual data were used to predict the probability of UTI at date of outpatient appointment in the previous cohort. Again, prediction probabilities and observed rates were compared.

## Results

### Patients’ characteristics

Between August 2012 and September 2014, 6,763 patients were screened for UTI when presenting at our transplant outpatient clinics. 102 RTx patients fulfilled the inclusion criteria of this study. The patients included were between 19 and 80 years of age (55.4±15.1) and 82 (80%) were female (Table 1). The 102 control RTx patients were between 22 and 79 years of age (50.6±13.5) and 38 (37%) were female.

**Table 1:** Patients' characteristics.

	UTI (n = 102)	no UTI (n = 102)	*P*-value
age (yr)	55.4±15.1	50.6±13.5	0.018^a^
weight (kg)	70.1±13.9	78.0±15.3	<0.001^a^
height (m)	165.5±8.5	173.5±10.2	<0.001^a^
BMI (kg/m^2^)	24.8 (17.3–40.6)	25.2 (17.1–47.5)	0.648^c^
sex (m/f)	20 (20%) / 82 (80%)	64 (63%) / 38 (37%)	<0.001^b^
number of prior transplantations			
zero	84 (82%)	86 (84%)	0.803^b^
one	16 (16%)	13 (13%)	
two	2 (2%)	3 (3%)	
time from RTx until office presentation/UTI (months)	24.3 (0.8–307)	28.5 (0.4–279)	0.677^c^
preemptive transplantation	7	10	0.614^b^
living donor transplantation	25 (25%)	41 (40%)	0.024^b^
ESP	19 (19%)	9 (9%)	0.066^b^
ABOi	5 (5%)	8 (8%)	0.568^b^
cumulative time on dialysis (months)	60 (3–225)	52 (2–243)	0.134^c^
CIT (h)	8.5 (1.1–35.0)	8.0 (1.0–30.0)	0.213^c^
WIT (min)	30.5±8.6	31.6±7.6	0.320^a^
donor data			
donor age	50.4±17.9	52.9±12.8	0.251^a^
donor sex (m/f)	61 (60%) / 41 (40%)	45 (44%) / 57 (56%)	0.035^b^

Variables are reported as absolute and relative frequencies, mean ± standard deviation or median (minimum-maximum);

^a^ t-test for independent groups;

^b^ Fisher's exact test;

^c^ Mann-Whitney U test; all patients were Western European descent.

UTI, urinary tract infection; BMI, body mass index; RTx, renal transplantation; ESP, European Senior Program; ABOi, ABO incompatible; CIT, cold ischemic time; WIT, warm ischemic time.

Univariate analysis revealed noticeably more females (p<0.001), a lower weight (p<0.001), a lower height (p<0.001) and an advanced age (p = 0.018) in UTI patients compared with the control group. Furthermore, the UTI group included fewer living donor transplantations (p = 0.024). Interestingly, more male kidney allografts were detected in the UTI cohort (p = 0.035). The logistic regression analysis revealed female sex (p<0.001), lower height (p = 0.040) and male kidney allograft (p = 0.029) as potential risk factors for the development of UTI (Table 2). Additionally, patients transplanted within the European Senior Program (ESP) had a higher risk for UTI (p = 0.026).

**Table 2:** Results of the multivariable logistic regression.

Independent variables	Odds ratio	95% Wald confidence limits		*P*-value
sex (male vs. female)	0.21	0.09	0.50	<0.001
log Tac C/D ratio at UTI (weight-adjusted) (x vs. x-1 units)	0.73	0.40	1.31	0.287
age (x vs. x-1 years)	1.00	0.98	1.03	0.785
height (x vs. x-1 cm)	0.96	0.92	1.00	0.040
living donor transplantation (yes vs. no)	0.57	0.26	1.27	0.170
ESP (yes vs. no)	3.86	1.18	12.69	0.026
donor sex (male vs. female)	2.11	1.08	4.10	0.029
AUC: 0.81 (95% CI 0.75–0.87)				

Results of the multivariable logistic regression of potential risk factors for UTI. Tac C/D ratio at UTI was log-transformed (natural logarithmic) to achieve equal intervals between C/D ratio units. P-values are from the Wald test; ESP, European Senior program; AUC, Area under the curve. *Equation3 for calculation of probability for UTI*: Prob(UTI) = exp(X)/(1+exp(X)), with X = 8.2674+1.5555·I(female)-0.3192·(logCDratio)+0.0043·(age)-0.0449·(height)-0.5602·I(ldt)+1.3512·I(ESP)-0.7444·I(female donor). I(female) = indicator of female sex, logCDratio = log Tac C/D ratio at UTI (weight-adjusted), age = age (years), height = height (cm), I(ldt) = indicator of living donor transplantation, I(ESP) = indicator of European Senior program, I(donor female) = indicator of female donor sex.

**Fig 1:**
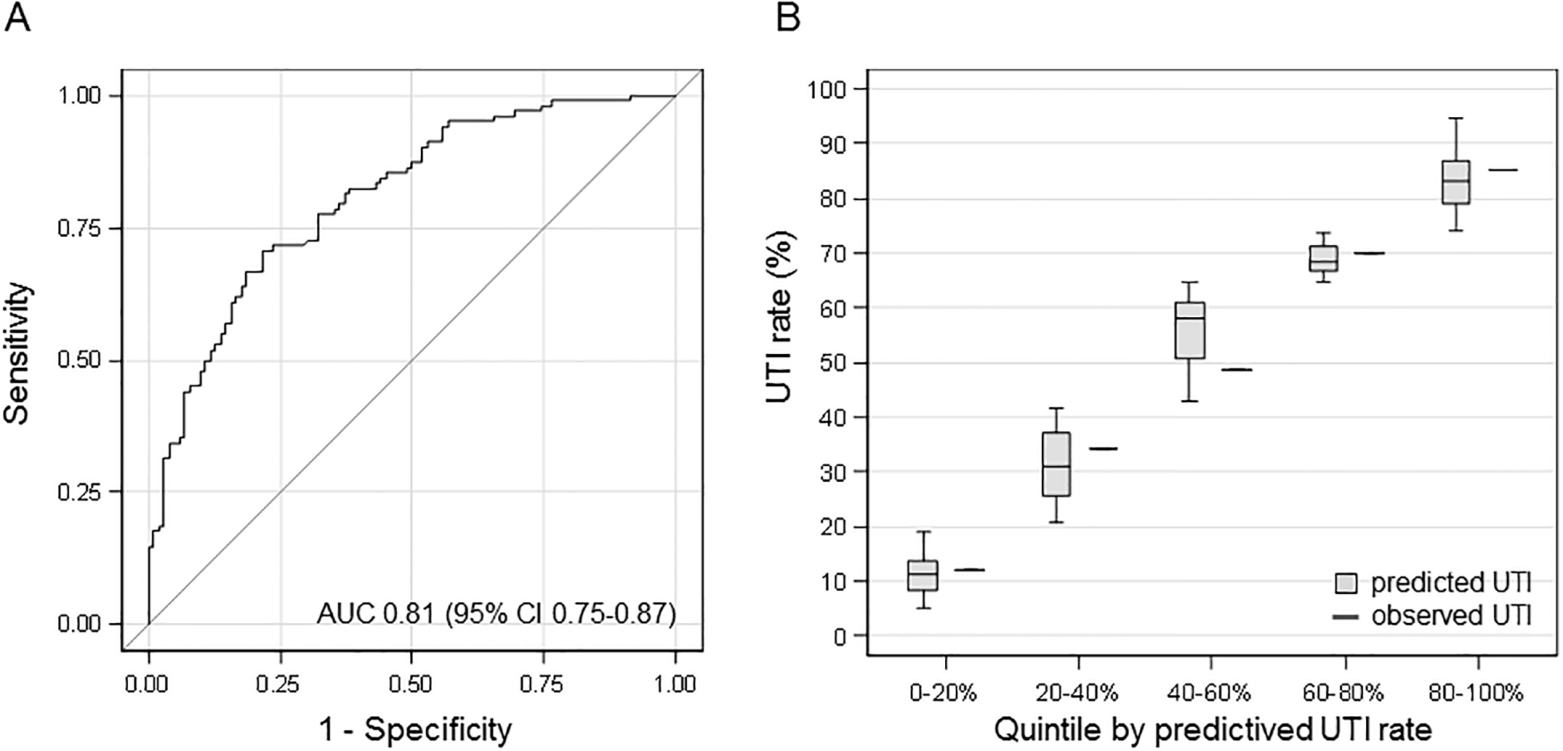
Correlation of predicted versus actual UTI rates in the actual cohort. **(A)** ROC curve of the logistic regression with area under the curve. **(B)** Predicted versus actual UTI rates. Prediction probabilities are calculated via Equation 3 (Table 2). Patients were classified into quintiles according to their individual predicted probability to suffer from UTI (boxes represent the IQR; whiskers indicate the minimum and maximum values, but are not longer than 1.5 times the IQR), which is plotted against the actual UTI rate for the quintile.

Reasons for end stage renal disease (ESRD) are given in S1 Table. The most frequent causes for ESRD were IgA nephropathy, autosomal dominant polycystic kidney disease (ADPKD) and chronic glomerulonephritis. Chronic pyelonephritis was more frequent in the UTI group compared to the control group (6% vs. 1%). The AUC of the multivariable model for the prediction of UTI was 0.81 (95% CI: 0.75–0.87; Fig 1).

### Drug doses and blood levels

Due to higher rates of previous infections in the UTI group, the administration of mycophenolate mofetil was stopped more often in UTI patients (number of patients on mycophenolate mofetil, p = 0.023, Table 3).

**Table 3:** Drug doses, blood levels, inflammatory markers and death.

	UTI (n = 102)	no UTI (n = 102)	*P*-value
Tac daily dose (mg)	4.0 (1.0–17.0)	4.0 (1.5–18.0)	0.457^a^
Tac blood trough level (ng/ml)	6.0 (1.5–25.8)	5.8 (2.8–14.8)	0.592^a^
Tac C/D ratio	1.4 (0.4–5.7)	1.5 (0.4–5.9)	0.451^a^
log Tac C/D ratio	0.3 (-1.0–1.7)	0.4 (-1.0–1.8)	0.451^a^
Tac C/D ratio (weight adjusted)	95.5 (25.4–464.4)	111.1 (26.1–519.2)	0.035^a^
log Tac C/D ratio (weight adjusted)	4.6 (3.2–6.1)	4.7 (3.3–6.3)	0.035^a^
Number of patients on everolimus	4	2	-
Everolimus daily dose (mg)	3.25 (2.5–5.0)	3	-
Prednisolone daily dose (mg)	5 (0–40)	5 (0–50)	0.758^a^
Number of patients on mycophenolate mofetil	62 (61%)	78 (77%)	0.023^b^
Mycophenolate mofetil daily dose (mg)	1000 (500–2000)	1000 (250–2000)	0.186^b^
250 mg	1 (1%)	0	
500 mg	17 (22%)	25 (40%)	
750 mg	5 (6%)	3 (5%)	
1000 mg	39 (50%)	27 (44%)	
1500 mg	9 (12%)	3 (5%)	
2000 mg	7 (9%)	4 (6%)	
Number of patients on mycophenolate sodium	12 (12%)	8 (8%)	0.481^b^
Mycophenolate sodium daily dose (mg)	720 (360–1080)	540 (360–720)	0.325^b^
360 mg	2	4	
720 mg	9	4	
1080 mg	1	0	
White blood count (x10^9^/L)	8.2 (3.1–27.6)	7.2 (3.2–17.7)	0.007^a^
C reactive protein (mg/dl)	0.5 (0.5–17.7)	0.5 (0.5–6.7)	<0.001^a^
Death	1	1	-

Variables are reported as absolute and relative frequencies, or median (minimum-maximum);

^a^ Mann-Whitney U test;

^b^ Fisher's exact test;

UTI, urinary tract infection; C/D, concentration/dose; Tac, tacrolimus

The weight-adjusted (p = 0.035) but not the non-weight adjusted Tac C/D ratio (p = 0.451) was lower in the UTI group (Table 3). However, in the logistic regression no influence of the log-transformed weight-adjusted C/D ratio on UTI development was detected. This might be explained by a dominating effect of female sex as a risk factor for UTI (Table 2). Females have a lower weight than males, which caused a univariate influence of the log-transformed weight-adjusted C/D ratio.

As expected, infection parameters such as white blood cell count and CRP were slightly elevated in UTI patients (Table 3). One patient in the UTI group died from urosepsis 12 months after inclusion in the study. In the control group, one patient died from Kaposi sarcoma 8 months after inclusion in the study.

### Renal function

At the time of presentation at our transplant center (UTI or no UTI), female patients who received a male kidney (male→female) revealed the highest eGFR values of all groups (Table 4). Due to large standard deviations, no statistical differences were found at this time point between donor/recipient sex groups within the UTI and no UTI group. After a follow-up of 12 months, analysis of different sex groups of both cohorts showed noticeable differences in eGFR values. While kidney function of female recipients was better than renal function of male recipients in general (p = 0.019 UTI group; p = 0.014 no UTI group), female recipients who received male grafts had the highest eGFR values in both groups (59±22 mL/min/1.73 m^2^ UTI group; 69±19 mL/min/1.73 m^2^ no UTI group).

**Table 4:** Sex-specific follow-up of renal function.

	UTI (n = 102)				*P*-value (within UTI)	no UTI (n = 102)				*P*-value (within no UTI)	*P*-value (UTI vs. no UTI)
	m→m	m→f	f→m	f→f		m→m	m→f	f→m	f→f		
number of patients at UTI/office presentation	12	49	8	33	-	27	19	36	20	-	<0.0001^a^
number of patients after 12 months	11	45	8	32	-	26	18	32	20	-	-
eGFR at UTI/office presentation	48±26	60±22	48±17	57±21	0.179^b^	53±23	64±19	50±18	56±19	0.059^b^	0.666^c^
eGFR after 12 months	40±25	59±22	52±32	58±19	0.019^b^	54±24	69±14	50±19	56±19	0.014^b^	0.876^c^
Δ eGFR	-4.5±10.2	0.9±17.5	4.5±18.2	2.5±10.5	0.362^b^	1.0±10.1	1.8±9.0	-2.4±9.6	-0.4±11.0	0.303^b^	0.205^c^

Patients with eGFR values at the time of UTI / office presentation and 12 months later. Abbreviations: eGFR, estimated glomerular filtrations rate; m, male; f, female; donor→recipient;

^a^ Fisher´s exact test for the comparison between UTI and no UTI for differences in donor/recipient sex group frequencies,

^b^ Kruskal-Wallis test for the comparison between donor/recipient sex groups within UTI and no UTI group;

^c^ Mann-Whitney U test for the comparison between UTI and no UTI.

Interestingly, there were no differences in the change of eGFR values (ΔeGFR) within the groups (UTI group p = 0.362 and no UTI p = 0.303) and between the groups (p = 0.205).

### Microbiology data

**Fig 2:**
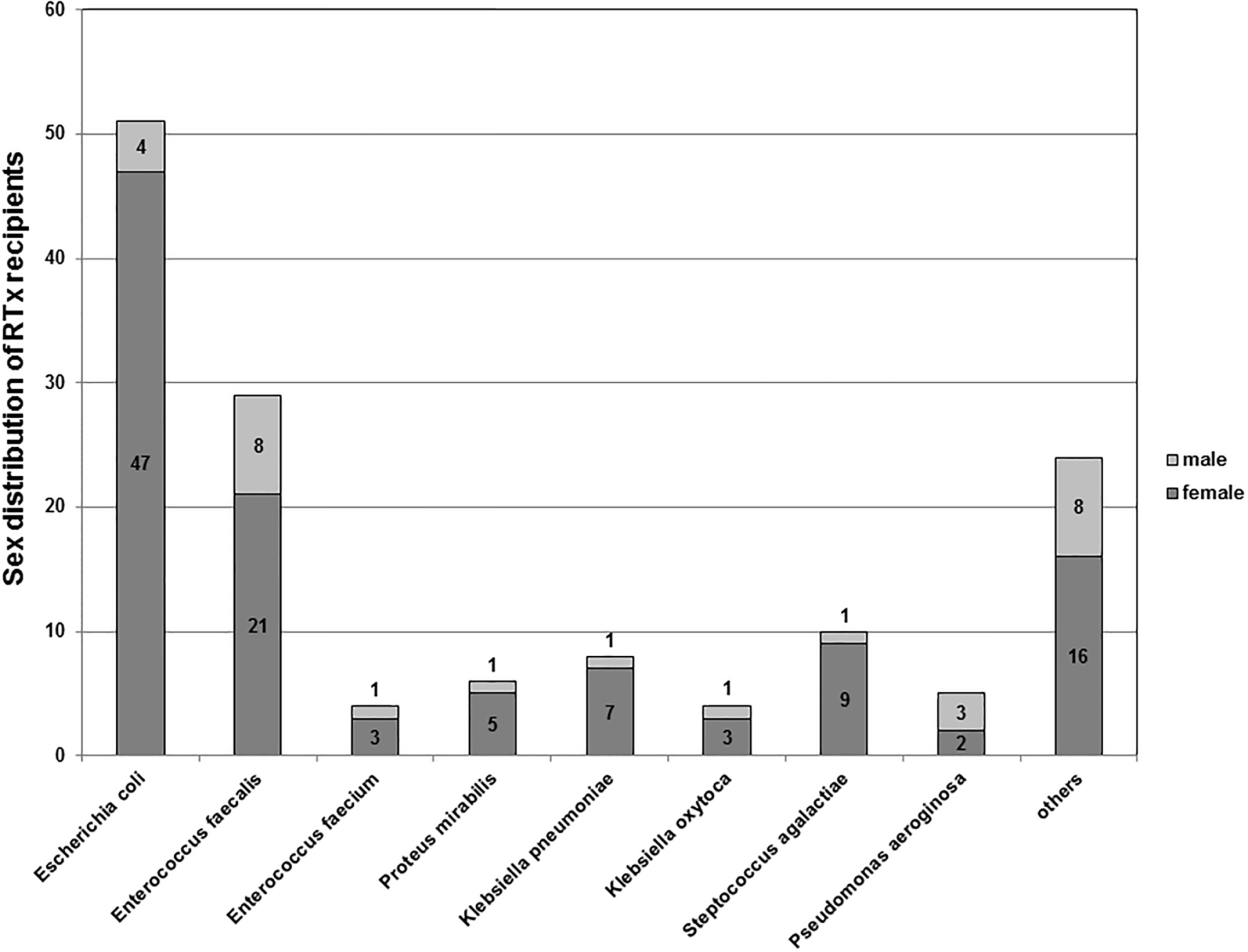
Pathogens detected in the urine of RTx recipients with UTI. *E*. *coli* and *Enterrococcus faecalis* were isolated in most of the cases.

At least one pathogen was isolated in the urine of 102 RTx patients. In further 26 patients two pathogens, in five three and in one four pathogens were isolated (Fig 2), respectively. The most frequently isolated pathogens were *Escherichia coli* (50%, n = 51), *Enterococcus faecalis* (28%, n = 29), *Streptococcus agalactiae* (10%, n = 10), *Klebsiella pneumoniae* (8%, n = 8). Other bacterial pathogens were found in 6% of isolates or less (Fig 2). *Candida albicans* was detected in 2% (n = 2) of urine cultures.

About 80% of UTI patients were females (Table 1). Interestingly, *E*. *coli* were detected in 46% (47/82) female and in 20% (4/20) male recipients. *E*. *faecalis* was found in 21% (21/82) female recipients and in 40% (8/20) male recipients, respectively. Due to the fact that in 29% of recipients more than one bacterial species was isolated from the urine, we did not perform an analysis of statistical differences between the groups.

**Fig 3:**
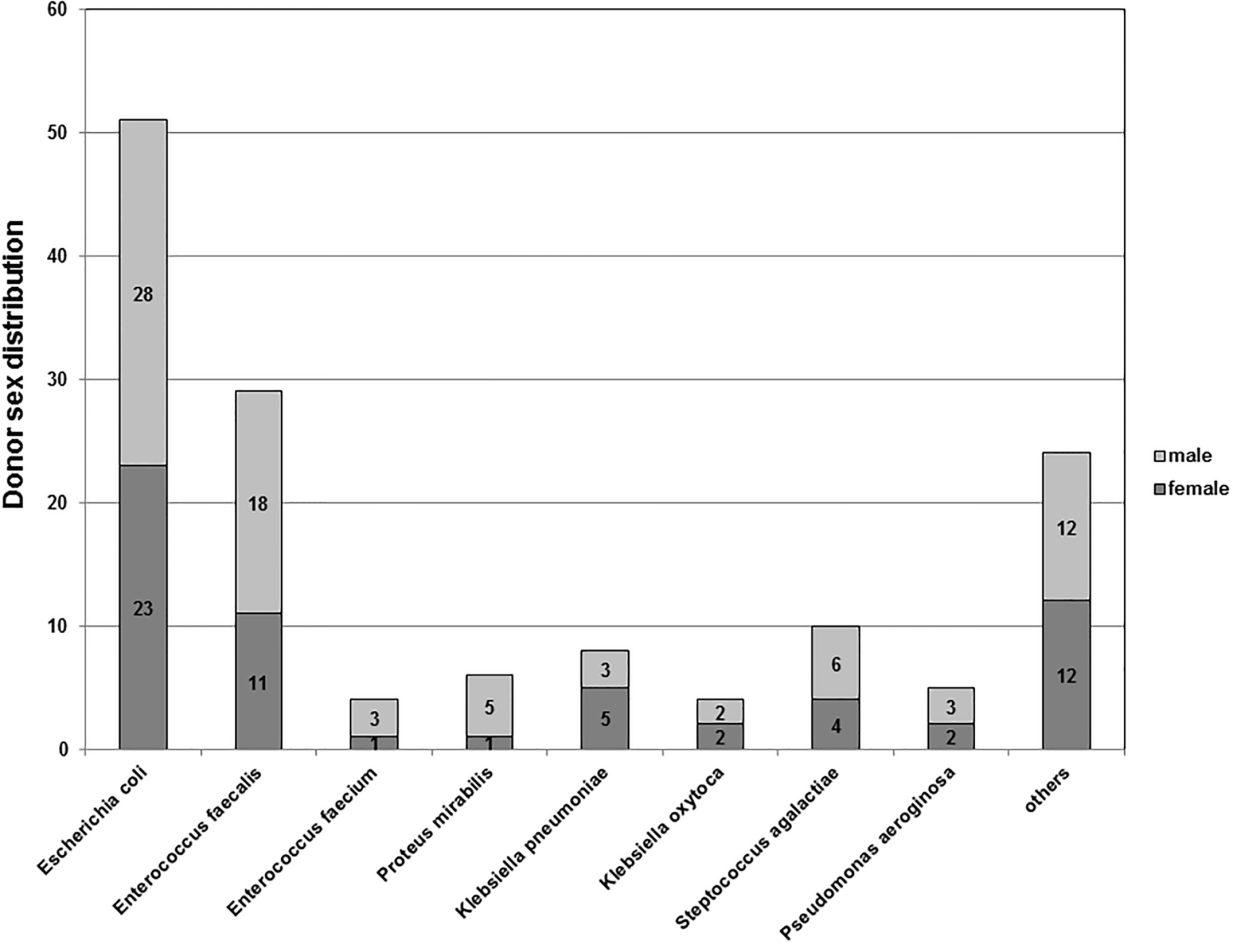
Distribution of bacteria isolated from different donor kidney sexes. While *E*. *coli* revealed an almost equal distribution in male and female urine (55%/45%), *Enterococcus faecalis* was more commonly isolated from the urine of male donor organs (62%).

In contrast, only 40% of donors were female. Thereby, almost equal proportions of *E*. *coli* were detected in the urine of organs from different sexes (28% (28/61) in female organs 23% (23/41) in male organs; Fig 3). *E*. *faecalis* was isolated from the urine in 18% male donor organs but only 11% from female organs.

### Analysis of the previous RTx cohort

In the previous cohort 112 of 311 patients, who received an initial immunosuppressive regimen with Tac, mycophenolate mofetil, prednisolone and basiliximab suffered from UTI. Median time between RTx and date of inclusion was 46 (0–94) months. S2 Table shows patients’ characteristics of the previous cohort. The UTI group in this cohort also included noticeably more female patients (p = 0.001) and older patients (p = 0.018) compared with the control group. Importantly, patients of the UTI group received more often male allografts (p = 0.012). The same logistic regression model used to analyze recent data was also applied on the previous cohort. In this analysis female sex (p = 0.066), advanced age (p = 0.010) and male donor sex (p = 0.072) were identified to be potential risk factors for the development of UTI (S3 Table).

For sensitivity analysis, the parameter estimates of logistic regression fitted for the actual data (Equation 3 shown in Table 2) were used to calculate prediction probabilities for UTI based on the observed data in the previous cohort. The predictive value (AUC) was 0.692 (95% CI: 0.598–0.787; S1 Fig). The predicted probabilities differ from the observed UTI rates in the quintile groups.

### Cytokine production by leukocytes

Initially, we tested whether leukocyte cytokine release differs between healthy subjects and RTx patients. Compared to RTx recipients, leukocytes from healthy donors showed higher levels of IL-8 at baseline (BL), while IL-1ß and S100A8/S100A9 releases were comparable between the groups (S4 Table). Additionally we found an increased BL production of IL-8 in females compared to males. Cytokine production (IL-8, IL-1β and S100A8/S100A9) increased noticeably in response to LPS-stimulation in all groups (p<0.0001, S4 Table).

Because the levels of spontaneous cytokine production by leukocytes were high, cytokine releases after LPS stimulation (LPS) were evaluated after subtracting the baseline values. Women showed a larger difference (LPS-BL) in S100A8/S100A9 production compared to men (p = 0.020).

## Discussion

We hypothesized that donor sex affects the occurrence of UTI after RTx. We found that UTI patients received more often male allografts than female allografts. This was observed in a retro- as well as in an essentially prospective cohort in our center. Therefore, we propose male kidney allograft sex as a new risk factor for UTI after RTx.

Female recipient sex had the greatest influence for the occurrence of UTI as shown in the univariate analysis and logistic regression in our study cohorts. This finding is in line with previous observations in RTx patients [1].

Second, the quality of organs seems to influence the occurrence of UTI. Many studies found an association between deceased donor allografts and an increased risk for UTI [1]. This association was confirmed in our study as we found less living donor transplantations than deceased donor allografts (univariate analysis) in the UTI group.

Third, in accordance with the identified UTI risk factors, advanced age, allografts of deceased donors and transplantation within the ESP (allografts and recipients ≥ 65 years of age) were associated with a higher risk for UTI (multivariable logistic regression).

As we identified male kidney allografts for the first time to be a possible risk factor for UTI irrespective of the recipient sex questions arise. Which factors distinguish male and female kidneys? Because kidney size correlates to body surface area, women usually have smaller kidneys and fewer nephrons than men resulting in a different kidney size [16]. However, a direct link between pure kidney size (probably with exception of patients with polycystic kidney diseases) and UTI development has not been shown. Notably, smaller kidney size itself might lead to inferior kidney function (nephron-dose theory) and kidney function has been shown to influence host response to infection, hence being a significant risk factor for infections including UTI [17, 18]. In our cohort, kidney function (eGFR) between the groups (male→male; male→female; female→male; female→female) was not noticeably different at the time of presentation at the outpatient clinics (UTI and no UTI patients). After a follow-up of 12 months, both cohorts showed noticeable differences in eGFR values. While male→female recipients had the highest eGFR values, the male→male and female→male group revealed the lowest eGFR. These findings might be biased by a considerably higher number of patients in the male→female group compared to the other groups. There were no differences in the change of eGFR values (ΔeGFR after 12 months vs. time of presentation) between the donor/recipient sex (within UTI and no UTI group) and between the UTI groups (UTI vs. no UTI).

According to our data, male kidney size is larger in average than female kidney size. Therefore, kidney function after male kidney donation might lead to a better eGFR than after female donation. This has been observed in some studies, while in others this could not be confirmed [19, 20]. Besides, other differences like sex-specific antigenicity (higher levels of HLA and non-HLA antigens in female kidneys, the H-Y antigen mismatch/H-Y effect observed upon transplantation of male kidneys to female recipients) or susceptibility to rejection are discussed and arouse interest to the immune response [5, 6, 20]. Both axes of the immune system, the innate as well as the adaptive one, are influenced by sex (hormones) [21]. As the immune response is critical for the defense against UTI and is needed for the clearance of infection, we analyzed leukocytes`cytokine response to stimulation of patients receiving a comparable immunosuppressive regimen [22, 23]. Because monocytes/macrophages and neutrophils are key cells in the host defense against uropathogenic bacteria, we analyzed important cytokines involved upon activation by LPS [23]. During early stages of UTI, a rapid influx of neutrophils into the bladder coincides with IL-8 release which aids in controlling infection [24, 25]. Interestingly, IL-8 production at BL was lower in immunosuppressed patients and men in general than in healthy controls and women (S4 Table). Analysis of donor sex did not reveal further difference. The alarmin S100A8/S100A9 is secreted by monocytes/macrophages and neutrophils after activation. It has locally cytokine-like functions, i.e. enhancing leukocyte recruitment to inflammatory sites and anti-microbial properties [26].

Difference in S100A8/S100A9 expression between LPS stimulation and baseline was only noticeably elevated in females compared to males (S4 Table). However, S100A8/S100A9 response was found not to be critical for infection control in a murine model of UTI [27]. Next, IL-1ß production was assessed as IL-1ß release by neutrophils is involved in chemotaxis and UTI clearance [28]. However, IL-1ß release increased after stimulation but was not different between groups.

As we could not detect a noticeable influence of the donor sex to the general cytokine response of leukocytes upon stimulation with LPS, the next important analysis would include extracted leukocytes from kidneys to assess the influence of the local micro milieu.

On the other hand soluble factors in the urine as well as factors concerning the urothelial structure of male and female urothelial cells including urethral glandular tissue should be analyzed.

Several anti-microbial peptides are excreted with the human urine [29]. It was shown that male and female urine proteomes are different [11, 12]. These analyses revealed e.g. relevant greater amounts of kallikrein-1 and male specific proteins (mainly prostate-origin proteins) in the male urine proteome. However, the fact that we identified male allograft sex independent of a prostate to be a risk factor for UTI leads to the question if the anti-microbial capacity of male urine is completed by prostate-derived or urethral glandular-derived peptides. Yet, appropriate prospective studies evaluating the risk for UTI in the long term after prostatectomy are missing. Therefore, to identify kidney-originated differences in excretion of anti-microbial peptides, the urinary proteome analysis should be performed in the transplant setting using e.g. urine from female recipients of male kidney without diuresis before transplantation to exclude an influence of the prostate and of urethral glands.

Renal function was unaffected one year after UTI and eGFR did not change considerably. In accordance to recent data from Bodro et al. allograft pyelonephritis is associated with an impairment of renal function while asymptomatic bacteriuria and acute cystitis are not [30].

Recently, we found an association between the Tac metabolism rate (C/D ratio), renal function and susceptibility to BK-virus infection after RTx [4, 14, 31]. A faster Tac metabolism rate negatively impacted on renal function and BK-virus infection rate. Therefore, we tested the hypothesis if Tac metabolism is also associated with UTI frequency. We could not observe a statistically noticeable association between both variables. This is in line with the fact that Tac as a calcineurin-inhibitor mainly affects T-cell viability while the host defense to UTI is mainly based on innate immune cells.

Finally, we analyzed the pathogen pattern found in our UTI patients. In accordance to the literature *E*. *coli* was the most common identified bacteria followed by infections due to *Enterococcus faecalis* [32].

We questioned if the pathogen pattern differs between male and females and if the allograft sex is relevant to this. In this regard Linhares et al. showed that e.g. *Pseudomonas aeruginosa* infection is more relevant to men than to women [33]. In our study, *E*. *coli* was mainly found in the urine of female recipients (92%), while *Enterococcus faecalis* was more common in organs from male donor kidneys (62%). The reasons for the sex-specific etiology of the UTI cases studied are unknown. Our findings, however, are in accordance with previously published data reporting on an increased frequency of *E*. *coli* isolates in female UTI patients, whereas other uropathogens incl. *Enterococcus* spp. were more frequently isolated from male patients with UTI [34–38].

This study has some limitations. This is an in part retrospective (patients`and donors`characteristics), single center study of mainly Western European descent patients. The occurrence of UTI was only measured at date of screening visit, regardless of the time since RTx. Information about former and later UTIs were not collected. So, this study can be seen as a type of cross-sectional case-control study with selected controls. Because of this kind of study type, estimation of incidence rates cannot be performed, and neither identification of risk factors nor prediction for the general development of UTI after RTx can be made. Therefore, prospective monitoring of the first occurrence of UTI since RTx and analyses using statistical time-to-event methods are necessary. However, despite the small number of patients in the retrospective analysis of the previous cohort, this led to a just reasonable accordance to the prediction model from the actual data.

It would be interesting whether other ethnic groups with different genetic backgrounds show the same pattern.

In conclusion, we first describe male kidney allograft sex to be a potential risk factor for UTI after RTx. In modern, individualized treatment regimens donors`and recipients`sex should be considered in the therapeutic approach.

## Supporting information

S1 Table Reason for end stage renal disease. (DOCX)

S2 Table Control data of a previous RTx cohort. (DOCX)

S3 Table Multivariable logistic regression analysis of the previous cohort. (DOCX)

S4 Table Cytokines. (DOCX)

S1 Fig Correlation of predicted versus actual UTI rates in the previous cohort based on the logistic regression model of the actual cohort. **(A)** ROC curve of the logistic regression with area under the curve. **(B)** Predicted versus actual UTI rates. Prediction probabilities are calculated via Equation 3 shown in Table 2. Patients were classified into quintiles according to their individual predicted probability to suffer from UTI (boxes represent the IQR; whiskers indicate the minimum and maximum values, but are not longer than 1.5 times the IQR), which is plotted against the actual UTI rate for the quintile.
(TIF)
