# Supplementary material for: Male kidney allograft recipients at risk for urinary tract infection?
Source: PLoS One. 2017 Nov 16;12(11):e0188262. doi: 10.1371/journal.pone.0188262 (PMC5690643; doi:10.1371/journal.pone.0188262)
Supplement: S1 Table — (DOCX) [file pone.0188262.s001.docx]

**S1 Table.** Reason for end stage renal disease.

|  | UTI (n=102) | no UTI (n=102) |
| --- | --- | --- |
| IgA nephropathy | 12 (12%) | 18 (18%) |
| ADPKD | 12 (12%) | 15 (15%) |
| minimal change GN | 1 | - |
| Alport-syndrome | 4 | 1 |
| chronic GN / unknown | 18 (18%) | 20 (20%) |
| interstitial nephritis | 8 (8%) | 6 (6%) |
| refluxnephropathy | 3 | 4 |
| FSGS | 5 (5%) | 6 (6%) |
| diabetic nephropathy | 8 (8%) | 4 |
| membranous GN | 1 | - |
| membranoproliferative GN | 2 | 1 |
| hydronephrosis | 3 | 1 |
| ANCA-associated nephropathy | 6 (6%) | 5 (5%) |
| aHUS | 1 | - |
| typical HUS | - | 2 |
| benign nephrosclerosis | 3 | 6 (6%) |
| lupus nephritis | 3 | 2 |
| chronic pyelonephritis | 6 (6%) | 1 |
| thrombotic microangiopathy (malignant hypertension) | 1 | - |
| septic shock | 2 | 1 |
| amyloidosis | 1 | - |
| CNI nephrotoxicity | - | 1 |
| nephronophthisis / congenital renal hypoplasia | 2 | 6 (6%) |
| Goodpasture syndrome | - | 2 |
| renal infarction | - | 1 |

Variables are reported as absolute and relative frequencies; ADPKD, autosomal dominant polycystic kidney disease; GN, glomerulonephritis; FSGS, focal segmental glomerulosclerosis; ANCA, antineutrophil cytoplasmic antibodies; aHUS, atypical hemolytic uremic syndrome; HUS, hemolytic uremic syndrome; CNI, calcineurin inhibitor.
