# Supplementary material for: Male kidney allograft recipients at risk for urinary tract infection?
Source: PLoS One. 2017 Nov 16;12(11):e0188262. doi: 10.1371/journal.pone.0188262 (PMC5690643; doi:10.1371/journal.pone.0188262)
Supplement: S2 Table — (DOCX) [file pone.0188262.s002.docx]

**S2 Table.** Control data of a previous RTx cohort.

|  | UTI (n=63) | no UTI (n=59) | p value |
| --- | --- | --- | --- |
| age (yr) | 55.6±12.4 | 49.9±13.8 | 0.018^a^ |
| height (cm) | 170±10 | 175±10 | 0.008^a^ |
| sex (male/female) | 25 (40%) / 38 (60%) | 41 (69%) / 18 (31%) | 0.001^b^ |
| living donor transplantation | 13 (21%) | 15 (25%) | 0.667^b^ |
| ESP | 13 (20%) | 12 (21%) | 1.0^b^ |
| Tac C/D ratio weight adjusted | 94.4 (27.6-380.0) | 95.8 (19.2-318.1) | 0.730^c^ |
| log Tac C/D ratio weight adjusted | 4.6 (3.0-5.8) | 4.6 (3.3-5.9) | 0.730^c^ |
| donor sex (male/female) | 37 (59%) / 26 (41%) | 21 (36%) / 38 (64%) | 0.012^b^ |

Variables are reported as absolute and relative frequencies, mean ± standard deviation or median (minimum-maximum); ^a^ t-test for independent groups; ^b^ Fisher's exact test; ^c^ Mann Whitney U test; all patients were Western European descent. UTI, urinary tract infection; RTx, renal transplantation; ESP, European Senior Program.
