# Supplementary material for: Male kidney allograft recipients at risk for urinary tract infection?
Source: PLoS One. 2017 Nov 16;12(11):e0188262. doi: 10.1371/journal.pone.0188262 (PMC5690643; doi:10.1371/journal.pone.0188262)
Supplement: S3 Table — (DOCX) [file pone.0188262.s003.docx]

**S3 Table.** Multivariable logistic regression analysis of the previous cohort.

| Independent Variables | Odds Ratio | 95% Wald | | p value |
| --- | --- | --- | --- | --- |
|  |  | Confidence Limits | |  |
| sex (male vs. female) | 0.30 | 0.08 | 1.08 | 0.066 |
| log Tac C/D ratio at UTI (weight-adjusted) (x vs. x-1 units) | 1.10 | 0.53 | 2.27 | 0.798 |
| age (x vs. x-1 years) | 1.06 | 1.01 | 1.10 | 0.010 |
| height (x vs. x-1 cm) | 0.99 | 0.93 | 1.05 | 0.726 |
| living donor transplantation (yes vs. no) | 1.22 | 0.42 | 3.49 | 0.716 |
| ESP (yes vs. no) | 0.52 | 0.14 | 1.93 | 0.330 |
| donor sex (male vs. female) | 2.10 | 0.94 | 4.73 | 0.072 |

AUC: 0.74 (95% CI 0.66-0.83).

Results of the multivariable logistic regression of potential risk factors for UTI. Tac C/D ratio at UTI was log-transformed (natural logarithmic) to achieve equal intervals between C/D ratio units. p values are from the Wald test; ESP, European Senior program; AUC, Area under the curve.
