# Supplementary material for: Male kidney allograft recipients at risk for urinary tract infection?
Source: PLoS One. 2017 Nov 16;12(11):e0188262. doi: 10.1371/journal.pone.0188262 (PMC5690643; doi:10.1371/journal.pone.0188262)
Supplement: S4 Table — (DOCX) [file pone.0188262.s004.docx]

**S4 Table.** Cytokines.

|  | healthy controls | RTx recipients | p value | males | females | p value |
| --- | --- | --- | --- | --- | --- | --- |
| IL-8 production (ng/ml) | | | | | | |
| baseline (BL) | 29.1 (26.2, 37.9) | 15.8 (12.0, 27.8) | 0.008 | 15.6 (10.4, 28.0) | 27.2 (15.2, 34.0) | 0.022 |
| LPS stimulation (LPS) | 189.6 (134.0, 228.7) | 112.9 (67.2, 221.6) | 0.052 | 107.8 (73.0, 212.9) | 154.4 (91.2, 271.1) | 0.157 |
| difference LPS-BL | 149.9 (109.4, 192.2) | 92.10 (52.0, 187.2) | 0.060 | 92.8 (56.8, 179.6) | 137.4 (70.8, 198.1) | 0.279 |
| IL-1β production (ng/ml) | | | | | | |
| baseline (BL) | 1.5 (0.9, 1.9) | 0.8 (0.0, 2.0) | 0.277 | 0.8 (0.0, 2.0) | 1.5 (0.0, 2.1) | 0.520 |
| LPS stimulation (LPS) | 3.2 (2.4, 4.7) | 3.2 (0.0, 6.4) | 0.936 | 3.5 (0.8, 6.5) | 3.0 (1.0, 5.1) | 0.557 |
| difference LPS- BL | 1.4 (0.7, 3.0) | 1.7 (0.0, 5.6) | 0.878 | 2.5 (0.2, 5.6) | 1.1 (0.3, 3.2) | 0.454 |
| S100A8/S100A9 production (ng/ml) | | | | | | |
| baseline (BL) | 6939 (4212, 9749) | 10314 (6145, 16343) | 0.073 | 10250 (5227, 16376) | 7867 (5855, 13776) | 0.418 |
| LPS stimulation (LPS) | 11896 (8283, 21610) | 13591 (7740, 23047) | 0.725 | 11945 (7661, 23036) | 15037 (9473, 23305) | 0.399 |
| difference LPS-BL | 4791 (2421, 8745) | 2233 (-368, 6656) | 0.063 | 1848 (-726, 4755) | 4803 (1845, 7059) | 0.020 |

Median (25% quantile, 75% quantile); p-values shown in the table are from the Mann-Whitney U test; p-values within the groups (i.e. healthy controls) BL vs. LPS are from the Wilcoxon signed rank test (all p<0.0001).

Median (25% quantile, 75% quantile); p-values shown in the table are from the Mann-Whitney U test; p-values within the groups (i.e. healthy controls) BL vs. LPS are from the Wilcoxon signed rank test (all p<0.0001).
