# Supplementary figures and images for: Male kidney allograft recipients at risk for urinary tract infection?
Source: PLoS One. 2017 Nov 16;12(11):e0188262. doi: 10.1371/journal.pone.0188262 (PMC5690643; doi:10.1371/journal.pone.0188262)

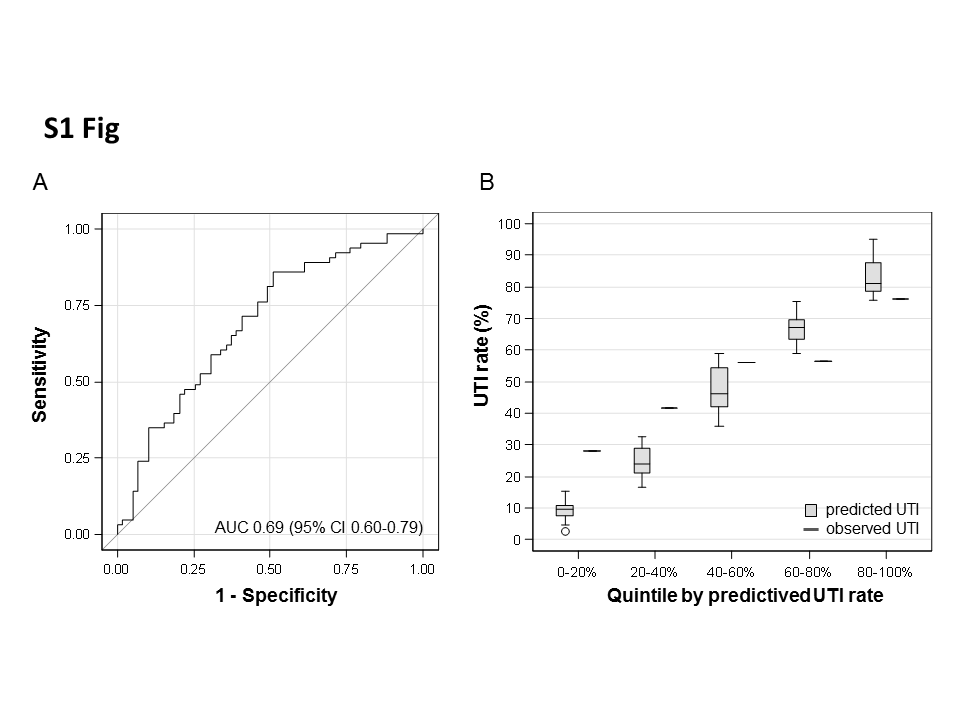

Supplement: S1 Fig — (A) ROC curve of the logistic regression with area under the curve. (B) Predicted versus actual UTI rates. Prediction probabilities are calculated via Equation 3 shown in Table 2. Patients were classified into quintiles according to their individual predicted probability to suffer from UTI (boxes represent the IQR; whiskers indicate the minimum and maximum values, but are not longer than 1.5 times the IQR), which is plotted against the actual UTI rate for the quintile. (TIF) [file pone.0188262.s005.tif]
